# Novel Fredericamycin Variant Overproduced by a Streptomycin-Resistant *Streptomyces* *albus* subsp. *chlorinus* Strain

**DOI:** 10.3390/md18060284

**Published:** 2020-05-28

**Authors:** Marta Rodríguez Estévez, Maksym Myronovskyi, Birgit Rosenkränzer, Thomas Paululat, Lutz Petzke, Jeanette Ristau, Andriy Luzhetskyy

**Affiliations:** 1 Pharmazeutische Biotechnologie, Universität des Saarlandes, 66123 Saarbrücken, Germany; marta.rodriguezestevez@uni-saarland.de (M.R.E.); maksym.myronovskyi@uni-saarland.de (M.M.); b.rosenkraenzer@mx.uni-saarland.de (B.R.); 2 Organische Chemie II, Universität Siegen, 57068 Siegen, Germany; paululat@chemie.uni-siegen.de; 3 BASF SE, 67056 Ludwigshafen, Germany; lutz.petzke@basf.de (L.P.); jeanette.ristau@basf.de (J.R.); 4 Helmholtz-Institut für Pharmazeutische Forschung Saarland, 66123 Saarbrücken, Germany

**Keywords:** antitumor, fredericamycin, overproduction, secondary metabolites, streptomycetes, streptomycin-resistant, type II PKS

## Abstract

Streptomycetes are an important source of natural products potentially applicable in the pharmaceutical industry. Many of these drugs are secondary metabolites whose biosynthetic genes are very often poorly expressed under laboratory cultivation conditions. In many cases, antibiotic-resistant mutants exhibit increased production of natural drugs, which facilitates the identification and isolation of new substances. In this study, we report the induction of a type II polyketide synthase gene cluster in the marine strain *Streptomyces* *albus* subsp. *chlorinus* through the selection of streptomycin-resistant mutants, resulting in overproduction of the novel compound fredericamycin C_2_ (**1**). Fredericamycin C_2_ (**1**) is structurally related to the potent antitumor drug lead fredericamycin A.

## 1. Introduction

The bacterial genus *Streptomyces* is well-known for producing a huge variety of bioactive secondary metabolites with potential pharmaceutical applications [1,2]. The genes responsible for their biosynthesis are generally clustered together. However, many of these biosynthetic genes are poorly or not expressed (silent) under laboratory cultivation conditions. Thus, the activation of silent secondary metabolite gene clusters is an essential step for the discovery of new natural drugs. A simple strategy to activate or upregulate the expression of these genes consists in screening for antibiotic-resistant mutants [3,4]. This approach is based on the work of Ochi and his team. They discovered that certain mutations in the *rpsL* and *rpoB* genes, which code for the ribosomal protein S12 and the β-subunit of RNA polymerase, respectively, lead to an altered gene product that confers resistance to streptomycin (*str* mutants) or to rifampicin (*rif* mutants), respectively. The mutations in these genes also increase the production of secondary metabolites in several *Streptomyces* strains [3,4,5,6,7]. Presumably, some of the *str* and *rpoB* mutations give rise to diverse metabolic changes which typically occur during the stringent response. The stringent response is triggered in *E. coli* [8] and other prokaryotic microorganisms [9,10] under amino acid starvation conditions by the signaling molecule, guanosine tetraphosphate (ppGpp). This molecule generates a series of physiological changes, including a significant decrease of protein synthesis [11], downregulation of rRNA transcription [12], and activation of stationary-phase metabolic processes, such as the expression of secondary metabolite biosynthetic genes [9]. Thus, it is suggested that the mutant gene products of *rpsL* and *rpoB* may be responsible for antibiotic overproduction, mimicking the role of ppGpp in the stringent response [3,4,5,6].

In previous studies, the marine strain *Streptomyces albus* subsp. *chlorinus* NRRL B-24108 has been shown to harbor genes for the production of several bioactive secondary metabolites, such as the herbicide albucidin or the antibiotic nybomycin [13,14]. Here, we report the induction of a type II polyketide synthase (PKS) gene cluster in *S. albus* subsp. *chlorinus*, leading to overproduction of the novel compound fredericamycin C_2_ (**1**). This substance is structurally related to fredericamycin C (**2**), a secondary metabolite first isolated in 1981, together with the compounds fredericamycin A and fredericamycin B, from the culture broth of *Streptomyces griseus* ATCC 49344 [15]. All fredericamycin variants known to date (A, B, C, C_1_, and E) share a similar structure that involves two aromatic moieties, which in the case of fredericamycins A and E are linked by a rare stereogenic spiro carbon center [16,17,18]. Fredericamycin A displays strong in vivo anticancer activity against several mammal tumor cell lines [19], and it has been patented as an effective treatment for certain types of cancer in humans [20]. Additionally, fredericamycins A, B, C, and C_1_ exhibit moderate antibacterial and antifungal activities [19]. In this study, we present the generation of the high-level streptomycin-resistant strain, *Streptomyces albus* subsp. *chlorinus* JR1, which overproduces the novel compound fredericamycin C_2_ (**1**). We also describe the mutation likely causing this phenotype and propose the initial biosynthetic steps of fredericamycin C_2_ (**1**), based on the gene cluster homology with that of fredericamycin A.

## 2. Results

### 2.1. High-Level Streptomycin-Resistant Mutant S. albus subsp. chlorinus Overproduces the Novel Compound Fredericamycin C_2_

**Figure 1:**
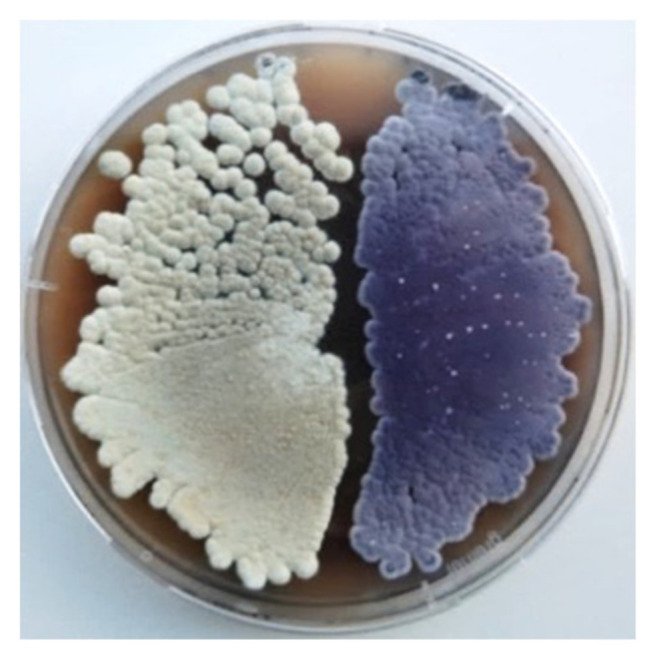
*Streptomyces albus* subsp. *chlorinus* NRRL B-24108 (left) and *Streptomyces albus* subsp. *chlorinus* JR1 (right) spores on MS agar medium.

**Figure 2:**
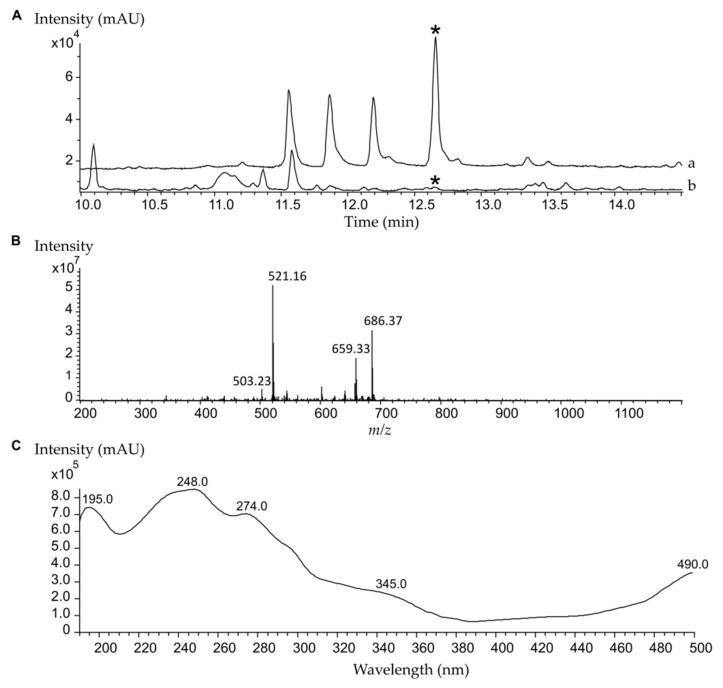
HPLC-MS analysis of crude extract from solid cultures of *S. albus* subsp. *chlorinus* JR1 (**a**) and its parental strain *S. albus* subsp. *chlorinus* NRRL B-24108 (**b**). (**A**) UV chromatogram. The asterisk (*) indicates the peak corresponding to fredericamycin C_2_ (**1**) at t_R_ = 12.7 min. (**B**) Mass spectrum associated with t_R_ = 12.7 min from the UV chromatogram displayed in (**A**). (**C**) UV spectrum of purified fredericamycin C_2_ (**1**).

The strain *S. albus* subsp. *chlorinus* NRRL B-24108 was cultured in MS agar medium containing increasing concentrations of streptomycin. After several rounds of selection, we isolated a streptomycin-resistant colony that displayed a distinctive violet color when cultured on a solid medium, in contrast to the characteristic white color of the parental strain (Figure 1). We named the mutant strain *S. albus* subsp. *chlorinus* JR1. While the growth of the wild type of *S. albus* subsp. *chlorinus* was inhibited by a streptomycin concentration of 50 μg/mL, the mutant strain was able to grow in the presence of up to 200 μg/mL of antibiotic (Table S1). The metabolic profiles of both strains were analyzed using liquid chromatography and mass spectrometry, revealing the presence of a peak at t_R_ = 12.7 min that corresponds to an [M + H]^+^ ion of *m/z* 521.107 and displays UV absorption signals at λ_max_ 195, 248, 274, 345, and 490 nm (Figure 2). The peak area revealed an over 50-fold greater production of the corresponding compound by *S. albus* subsp. *chlorinus* JR1 compared to the parental strain’s yield (Table S2, Figure S1). The search of this mass in a natural product database yielded no coincidences, suggesting a potentially undescribed metabolite overproduced by *S. albus* subsp. *chlorinus* JR1. The compound was extracted from a 2 l solid culture of *S. albus* subsp. *chlorinus* JR1 and purified by normal phase chromatography through a silica column followed by reverse phase chromatography.

**Figure 3:**
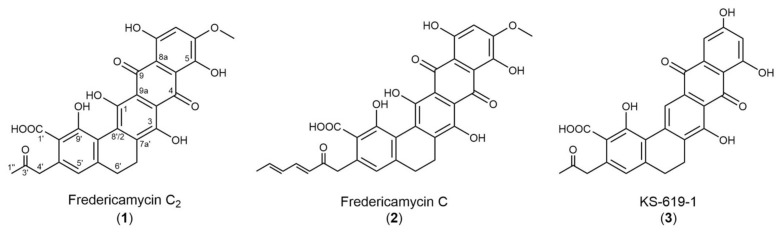
Structures of fredericamycin C_2_ (**1**), fredericamycin C (**2**) and KS-619-1 (**3**).

The molecular formula of the substance was determined to be C_27_H_20_O_11_ based on high-resolution MS (ESI) showing the quasi molecular ion *m/z* 521.107 ([M + H]^+^, calculated for C_27_H_21_O_11_). In the proton NMR spectrum, two broad multiplets are visible at δ_H_ 2.63 (t, 6’-H_2_) and 2.79 (t, 7’-H_2_) ppm, which are two neighbored methylene groups. All other signals are singlets: One methoxy group at δ_H_ 3.94 (6-OCH_3_), one additional methylene group at δ_H_ 4.06 (4’-H_2_), one methyl group at δ_H_ 2.17 (1’’-H_3_), and two methines at δ_H_ 6.52 (5’-H) and 6.88 (7-H). Moreover, broad singlets at δ_H_ 5.5, 12.8, and 13.2 ppm indicate hydroxyl groups to be present in the molecule (Table 1). Typical quinone carbonyl signals at δ_C_ 186.9 (C-4) and 187.6 (C-9) are visible in the ^13^C-NMR spectrum. A signal at δ_C_ 169.3 ppm (C-1’) shows an acid functionality which is attached at C-8’ proven from an HMBC (Heteronuclear Multiple Bond Coherence) correlation C-1’/ 5’-H. A very weak signal at δ_H_ 204.1 ppm (C-3’) shows a ketone supported by an HMBC cross-peak C-3’/1’’-H_3_ (Table 1). This ketone is part of a propan-2-on sidechain which is attached at C-6’ based on HMBC signals C-6’/4’-H_2_, C-6’/1’’-H_3_, C-5’/4’-H_2_, and C-7’/4’-H_2_. The HMBC signal C-6/6-OCH_3_ indicates the methoxy group to be attached at C-6 next to the aromatic proton 7-H. One of the rings contains a CH_2_-CH_2_ moiety (C-6’-C7’) which is in ring D proven from HMBC signals C-3/7’-H_2_, C-7a’/7’-H_2_, C-8’/7’-H_2_, C-7a’/6’-H_2_, and C-5a’/6’-H_2_ (Figure S2). Comparison to literature shows high similarity of the new compound named fredericamycin C_2_ (**1**) to fredericamycin C (**2**) [17], which differs only in the sidechain attached at C-6’ (fredericamycin C) and to KS-619-1 (**3**) [21] with a different substitution pattern of the pentacyclic ring system (KS-619-1) (Figure 3).

### 2.2. Fredericamycin C_2_ Is Biosynthesized by a Type II PKS Gene Cluster

**Figure 4:**
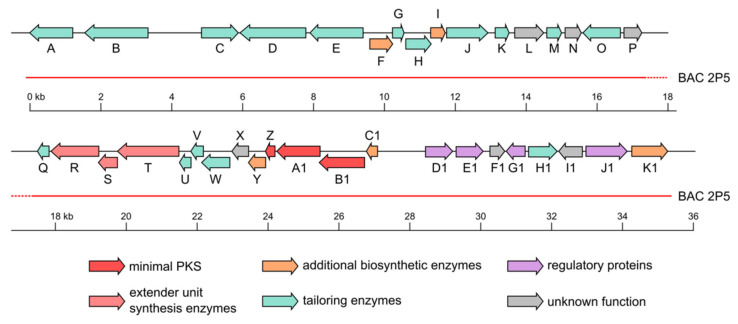
Map of the genes encoded in BAC 2P5 isolated from a genomic library of *S. albus* subsp. *chlorinus*. Characters from A to K1 indicate the corresponding *c2fdm* gene described in Table 2.

Fredericamycin C_2_ (**1**) is structurally related to other fredericamycin variants, such as C and A, which are biosynthesized by a type II PKS gene cluster [22]. This suggests the involvement of a type II PKS system in the production of fredericamycin C_2_ (**1**). Although a subclass of type I PKSs (iterative type I PKSs) have also been reported to synthesize aromatic compounds [23,24,25,26], their products are structurally smaller and simpler than the complex multicyclic metabolites produced by type II PKSs [27]. The pentacyclic aromatic polyketide structure of fredericamycin C_2_ (**1**) (Figure 3) further supports the assumption of type II PKS genes involved in its biosynthesis. The genome of *S. albus* subsp. *chlorinus* was screened for secondary metabolite genes, revealing the presence of one type II PKS gene cluster. Based on protein BLAST analysis, we assigned the genes putative functions, which are summarized in Table 2. To test whether the expression of these genes leads to fredericamycin C_2_ (**1**) production, BAC 2P5 containing the identified type II PKS cluster was isolated from a genomic library of *S. albus* subsp. *chlorinus* NRRL B-24108 and transferred via intergeneric conjugation into the heterologous host *Streptomyces albus* Del14. HPLC-MS analysis of the extract from the resulting ex-conjugant *S. albus* 2P5 revealed the presence of a peak with identical retention time and *m/z* to those of fredericamycin C_2_ (**1**), demonstrating that the type II PKS cluster from *S. albus* subsp *chlorinus* is responsible for fredericamycin C_2_ (**1**) biosynthesis (Figure S3, Figure 2). BAC 2P5 comprises a 35 kb genomic region containing a total of 37 open reading frames (ORFs), 10 of which share homology at protein level with the fredericamycin A gene cluster from *S. griseus* (Table 2, Figure 4), which is also responsible for fredericamycin C (**2**) biosynthesis [22]. The homologue genes include those coding for the minimal PKS ketosynthase subunits (KS_α_ and KS_β_), two polyketide cyclases, four tailoring enzymes, a transcriptional regulator, and a protein of unknown function. The gene similarity with fredericamycin A cluster, together with the heterologous expression results (Figure S3), indicates the relevance of these genes in fredericamycin C_2_ (**1**) biosynthesis.

### 2.3. Screening for the Mutation Causing Fredericamycin C_2_ Overproduction and Streptomycin Resistance in S. albus subsp. chlorinus JR1

Frequently, streptomycin resistance results from a point mutation in the *rpsL* gene, which codes for the ribosomal protein S12 [3,4,28,29,30]. Following genome sequencing of *S. albus* subsp. *chlorinus* JR1, the resulting reads were mapped to the genome of the wild type strain and single nucleotide mutations were searched in the sequence corresponding to the *rpsL* gene. No point mutations were found in this sequence, indicating that the mutation responsible for streptomycin resistance in *S. albus* subsp. *chlorinus* JR1 is located elsewhere in the genome. A total of fifteen point mutations were detected in the genome of *S. albus* subsp. *chlorinus* JR1 (Table S3). A nucleotide insertion was detected within the coding sequence of the *jag* gene (SACHL2_00217; position 3714884) that codes for a single-stranded DNA binding protein. This gene partially overlaps with the adjacent downstream gene *rsmG* (SACHL2_00216), indicating their co-transcription in a bicistronic operon. The insertion of a cytosine nucleotide in the sequence of *jag* creates a premature stop codon that truncates the protein translation, also affecting the expression of the co-translating *rsmG* gene. *rsmG* codes for a 16S rRNA methyltransferase, and it has been reported that point-nonsense mutations in its sequence, as well as the deletion of this gene, lead to increased resistance to streptomycin and enhanced production of secondary metabolites in different bacterial strains [31,32]. Therefore, we believe that a point mutation in the *jag* gene is responsible for high-level streptomycin resistance and upregulation of fredericamycin C_2_ (**1**) biosynthetic gene expression in *S. albus* subsp. *chlorinus* JR1 by preventing the translation of the *rsmG* gene.

### 2.4. Biological Activity of Fredericamycin C_2_

Fredericamycin C_2_ (**1**) was tested for antibacterial activity against a Gram-positive (*Bacillus subtilis*) and two Gram-negative strains (*Escherichia coli* and *Pseudomonas putida*) through disk diffusion test. The new fredericamycin variant displays a growth inhibition zone against *P. putida* at a minimal concentration of 2.5 mg/mL (Figure S4). Fredericamycin C_2_ (**1**) shows no inhibitory activity against the growth of *B. subtilis* and *E. coli*.

## 3. Discussion

Fredericamycin variants constitute a family of aromatic polyketides with significant toxicity against tumor cells as well as moderate antibiotic and antifungal activity. Here, we present the novel variant fredericamycin C_2_ (**1**), which is overproduced by the strain *S. albus* subsp. *chlorinus* JR1, a spontaneous streptomycin-resistant mutant derived from *S. albus* subsp. *chlorinus* NRRL B-24108. We suggest that a mutation in the *jag* gene affecting the translation of the adjacent gene *rsmG* is responsible for the phenotype of the mutant strain. The frame shift originated by the point insertion putatively generates a truncated non-functional Jag protein. In previous studies, deletion of a *jag* homologue in *Streptococcus pneumoniae* led to retarded growth and smaller cell size compared to the wild type strain, indicating that Jag is likely involved in cell division [33]. However, no association of *jag* deletion with increased antibiotic resistance or induction of secondary metabolite production has been reported before. The point mutation in the *jag* gene has a polar effect on the overlapping gene *rsmG*, preventing its transcription. The enhanced production of fredericamycin C_2_ (**1**), as well as the increased streptomycin resistance observed in *S. albus* subsp. *chlorinus* JR1, is most likely derived from the lack of RsmG function. This is consistent with previous studies where *rsmG* deletion mutants showed higher resistance to streptomycin and improved yields of secondary metabolites at a late-growth phase [34,35]. *rsmG* encodes a methyltransferase that catalyzes the methylation of 16S rRNA at the residue G527 (*E. coli* numbering). This residue, together with C526 and the S12 protein, interacts with the antibiotic streptomycin [36]. These interactions tend to stabilize the tRNA-mRNA tandem, which affects the proof-reading process and results in misreading of the genetic code [37]. The absence of RsmG would generate 16S rRNA molecules non-methylated at residue G527, causing a weaker binding to streptomycin and making the strain resistant to the antibiotic. Although the mechanism by which the lack of 16S rRNA methyltransferase may induce the expression of fredericamycin C_2_ (**1**) biosynthetic genes in *S. albus* subsp. *chlorinus* JR1 remains unknown, we hypothesize that the mutant experiences an increased protein synthesis rate at a stationary phase, as it has been previously observed [34,35]. The increased protein synthetic activity leads to expression of both pleiotropic and pathway-specific regulatory proteins, which eventually enhance the transcription of poorly expressed secondary metabolite gene clusters. Several attempts to complement the *jag* and *rsmG* gene functions in *S. albus* subsp. *chlorinus* JR1 resulted in no recombinant colonies, suggesting the genetic intractability of the strain (data not shown).

**Figure 5:**
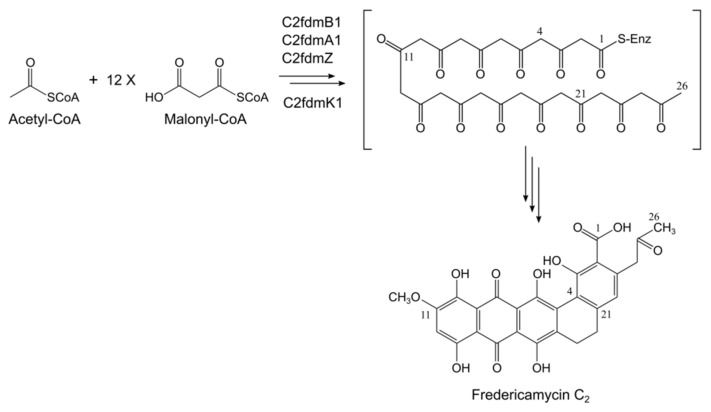
Proposed early biosynthesis steps of fredericamycin C_2_ (**1**) in *S. albus* subsp. *chlorinus*.

The structure of fredericamycin C_2_ (**1**) presented in this paper only differs from that of fredericamycin C (**2**) in the polyketide chain length. While fredericamycin C (**2**) backbone consists of 30 carbon atoms, fredericamycin C_2_ (**1**) contains a C_26_ polyketide chain (Figure 3). Fredericamycin C (**2**) biosynthesis begins with the generation of a C_6_ primer unit (hexadienyl-ACP) by the PKS initiation module. This starter unit is then transferred to the elongation module, which presumably catalyzes the sequential decarboxylative condensation of 12 malonyl-CoA molecules, delivering a C_30_ polyketide chain [22,38]. In the case of fredericamycin C_2_ (**1**), we propose that acetyl-CoA functions as the starter unit, which is elongated by the polyketide synthase through successive incorporation of malonyl-CoA extender units (Figure 5). This process is most likely catalyzed by the minimal PKS enzymes encoded by the genes *c2fdmB1*, *c2fdmA1*, and *c2fdmZ*, and the ACP S-malonyltransferase encoded by *c2fdmK1* (Table 2). The resulting C_26_ polyketide chain is subsequently modified by tailoring enzymes to eventually yield the product fredericamycin C_2_ (**1**) (Figure 5).

## 4. Materials and Methods

### 4.1. General Experimental Procedures

All strains and BACs (bacterial artificial chromosomes) used in this work are listed in Table S4. *Escherichia coli* strains were cultured in LB medium [39]. *Streptomyces* strains were grown on soy flour mannitol agar (MS agar) [40] for sporulation and conjugation and in liquid tryptic soy broth (TSB; Sigma-Aldrich, St. Louis, MO, USA). For metabolite expression, liquid DNPM medium (40 g/L dextrin, 7.5 g/L soytone, 5 g/L baking yeast, and 21 g/L MOPS, pH 6.8) or MS agar were used. The antibiotics kanamycin, apramycin, and nalidixic acid were supplemented when required.

### 4.2. Isolation and Manipulation of DNA

BAC extraction from a *Streptomyces albus* subsp. *chlorinus*-constructed genomic library (Intact Genomics, St. Louis, MO, USA), DNA manipulation, *E. coli* transformation, and *E. coli*/*Streptomyces* intergeneric conjugation were performed according to standard protocols [39,40,41]. Plasmid DNA was purified with the BACMAX™ DNA purification kit (Lucigen, Middleton, WI, USA). Restriction endonucleases were used according to manufacturer’s recommendations (New England Biolabs, Ipswich, MA, USA).

### 4.3. Strain Generation, Metabolite Extraction, and Analysis

The spontaneous streptomycin-resistant mutant *Streptomyces albus* subsp. *chlorinus* JR1 was obtained after several rounds of selection of streptomycin-resistant colonies growing on MS agar medium containing increasing concentrations of the antibiotic. MICs of streptomycin were determined by spreading spores on MS agar plates containing 50, 100, and 200 μg/mL of streptomycin. Metabolites were extracted from the agar with ethyl acetate acidified with 100% acetic acid up to pH = 2.0, evaporated and dissolved in methanol. One μL of extract was separated using a Dionex Ultimate 3000 UPLC (Thermo Fisher Scientific, Waltham, MA, USA), a 10-cm ACQUITY UPLC® BEH C18 column, 1.7 μm (Waters, Milford, MA, USA), and a linear gradient of 0.1% formic acid solution in acetonitrile against 0.1% formic acid solution in water from 5% to 95% in 18 min at a flow rate of 0.6 mL/min. Samples were analyzed using an Orbitrap speed mass spectrometer (Thermo Scientific, Waltham, MA, USA). Data were collected and analyzed with the Thermo Xcalibur software, version 3.0 (Thermo Scientific, Waltham, MA, USA). The monoisotopic mass was searched in a natural product database.

### 4.4. Fredericamycin C_2_ Purification and Quantification

*S. albus* subsp. *chlorinus* JR1 was grown for 8 days at 28 °C on 50 Petri dishes, each containing 40 mL of DNPM agar. Fredericamycin C_2_ (**1**) was extracted from the solid agar using ethyl acetate acidified with 100% acetic acid up to pH = 2.0. The crude extract was first separated by normal phase chromatography on a prepacked silica cartridge (Biotage, Uppsala, Sweden) using hexane (solvent A), chloroform (solvent B), ethyl acetate (solvent C), and methanol (solvent D) (1:1:1:1) as the mobile phase, in a linear gradient from 0% to 100% of each pair of solvents (A-B, B-C, and C-D). Fractions containing fredericamycin C_2_ (**1**) were detected by LC-MS analysis, pooled together, and further fractionated by semi-preparative HPLC (Dionex UltiMate 3000, Thermo Fisher Scientific, Waltham, MA, USA) using a C18 column (Synergi 10 μm, 250 × 10 mm; Phenomenex, Aschaffenburg, Germany) and a 0.1% formic acid solution in acetonitrile as the mobile phase in a linear gradient. UV spectra were recorded with a DAD detector at 274 nm and 525 nm. Finally, 0.8 g of fredericamycin C_2_ (**1**) was collected in a single fraction. For quantification, a calibration curve with different fredericamycin C_2_ (**1**) concentrations was constructed (Figure S1).

*Fredericamycin C*_2_ (**1**)*:* Violet, amorphous solid; *m/z* 521.1077 [M + H]^+^ (calculated for C_27_H_21_O_11_, 521.1084); UV λ_max_ (MeOH) 195, 248, 274, 345, 490 nm; ^1^H and ^13^C-NMR data, Table 1 and Supplementary Information Figures S2, S5–S10.

### 4.5. H-NMR Spectroscopy

NMR data were measured using a Varian VNMR-S600 spectrometer equipped with 3 mm triple resonance inverse and 3 mm dual broadband probes. Fredericamycin C_2_ (**1**) samples were dissolved in 150 µL DMSO-d6/Pyridine-d5 95:5 and measured at 35 °C. The residual solvent signal of DMSO was used as an internal reference.

### 4.6. Antimicrobial Susceptibility Test

Disk diffusion tests were performed according to [42]. Ten mL of LB soft agar (10 g/L tryptone, 10 g/L NaCl, 5 g/L yeast extract, 7 g/L agar) was inoculated with the strains *Escherichia coli* GB2005, *Bacillus subtilis* ATCC 6633, or *Pseudomonas putida* KT2440, and poured on LB agar plates. Five paper disks (Macherey and Nagel, Düren, Germany) were coated with 10, 5, 2.5, 0.5, or 0.25 mg/mL of fredericamycin C_2_ (**1**) solved in methanol and placed onto the solidified soft agar. An additional disk loaded with methanol was used as a negative control and the antibiotics nalidixic acid, ampicillin, and chloramphenicol (50 μg/mL, respectively) were used as positive controls. The plates were incubated at 28 °C overnight.

### 4.7. Genome Sequencing, Genome Assembly, and Analysis

*S. albus* subsp. *chlorinus* JR1 was sequenced using an Illumina MiSEQ library with 301-bp inserts (Illumina, San Diego, CA, USA). *S. albus* subsp. *chlorinus* JR1 strain genome assembly has a total of 57 contigs, and 5 final scaffolds—7,539,766 bp; 63,833 bp; 4,962 bp; 2,694 bp; and 2,548 bp (assembled with Newbler version 2, Roche, Basel, Switzerland). Sequencing reads coverage against the *S. albus* subsp. *chlorinus* B-24108 genome (Genbank accession number VJOK00000000) was examined with Geneious, version 11.0.3 (Biomatters Ltd., Auckland, New Zealand).

### 4.8. Genome Mining and Bioinformatics Analysis

The genome of *S. albus* subsp. *chlorinus* was screened for secondary metabolite biosynthetic gene clusters using the antiSMASH [43] online tool (https://antismash.secondarymetabolites.org/#!/start). Analysis of genetic data was performed using the Geneious software, version 11.0.3 (Biomatters Ltd., Auckland, New Zealand) [44].

## 5. Conclusions

Here, we demonstrate the significance of inducing poorly expressed secondary metabolite gene clusters for the identification of new microbial natural products. A mutation in the genome of the strain *S. albus* subsp. *chlorinus* JR1 obtained by selection of streptomycin-resistant colonies has led to overproduction and the discovery of the so far undescribed compound fredericamycin C_2_ (**1**). This secondary metabolite expands the structural variability of the fredericamycin family, whose most prominent member, fredericamycin A, is a potent antitumor drug. Screening for antibiotic-resistant strains represents a simple and inexpensive approach that has enabled the improvement of secondary metabolite production in the genetically intractable bacterial strain *S. albus* subsp. *chlorinus*.

## Figures and Tables

**Table 1:** NMR data of fredericamycin C_2_ (**1**) (600/150 MHz, DMSO-d6/Pyridine-d5 95:5, 35 °C, solvent (DMSO) as internal reference).

Pos. ^a^	δ_C_	δ_H_ (*J* Hz)	COSY ^b^	HMBC ^b^	ROESY ^b^
9’	164.3			5’-H	
9a’	115.3			4’-H_2_, 5’-H	
3’	140.7			4’-H_2_, (5’-H)	
5’	120.4	6.52 s	(4‘-H_2_)	^1^*J*, 4’-H_2_, (5’-H), 6’-H_2_	4’-H_2_, 6’-H_2_, (7’-H_2_)
5a’	144.3			5‘-H, 6‘-H_2_, 7‘-H_2_	
6’	28.8	2.63 br t (7Hz)	7’-H_2_	5’-H, 7’-H_2_	5’-H, 7’-H_2_
7’	21.2	2.79 br t (7Hz)	6’-H_2_	6’-H_2_	
7a’	137.3			6’-H_2_, 7’-H_2_	
3	152.5			7’-H_2_	
3a	112.2				
4	186.9				
4a	113.2			(7-H)	
5	148.8 ^c^			7-H	
6	157.6			6-OCH_3_, 7-H	
6-OCH_3_	56.4	3.94 s	6-H	^1^ *J*	7-H
7	105.8	6.88 s	6-OCH_3_	6-OCH_3_	6-OCH_3_
8	158.6			7-H	
8a	105.8			7-H	
9	187.6			7-H	
9a	114.4				
1	156.3				
2 = 8’	136.3			(5’-H), 7’-H_2_	
8a’	121.0			5’-H	
1’	169.3			5’-H	
4’	49.5	4.06 s	(5’-H, 1’’-H_3_)	5’-H, (1’’-H_3_)	5’-H, 1’’-H_3_
3’	(204.1) ^d^				
1’’	29.7	2.17 s			4’-H_2_
OH		13.22 br s 12.80 br s 5.5 br s			

**^a^** Numbering according to fredericamycin C (**2**); **^b^** weak signals in brackets; **^c^** from HMBC; **^d^** very weak signal in carbon NMR spectrum.

**Table 2:** Proposed functions of genes present in the type II polyketide synthase (PKS) cluster of *S. albus* subsp. *chlorinus* and homology with fredericamycin A gene cluster.

Gene	Size (aa)	Proposed Function	GenBank Homologue ^1^	Identity/Similarity (%)	Fredericamycin A Gene Cluster Homologue	Identity/Similarity (%)
*c2fdmA*	406	Cytochrome P450 oxygenase	WP_017596471.1	66/73	-	-
*c2fdmB*	594	Monooxygenase	WP_017596470.1	67/75	-	-
*c2fdmC*	337	O-methyltransferase	WP_043504920.1	42/55	-	-
*c2fdmD*	620	Asparagine synthase	WP_017596467.1	79/88	-	-
*c2fdmE*	497	Monooxygenase	WP_081620749.1	59/70	-	-
*c2fdmF*	216	Polyketide cyclase	REH43750.1	43/54	-	-
*c2fdmG*	107	Monooxygenase	WP_027732672.1	44/64	-	-
*c2fdmH*	237	3-ketoacyl-ACP reductase	WP_017596481.1	72/83	-	-
*c2fdmI*	134	Polyketide cyclase	WP_109361109.1	72/83	-	-
*c2fdmJ*	389	O-methyltransferase	WP_061257536.1	67/79	*fdmN*	56/70
*c2fdmK*	138	Oxidoreductase	WP_020573863.1	56/68	-	-
*c2fdmL*	263	Unknown	WP_017596478.1	56/71	-	-
*c2fdmM*	138	Oxidoreductase	WP_017596477.1	70/85	-	-
*c2fdmN*	150	Unknown	WP_017596476.1	76/89	-	-
*c2fdmO*	358	O-methyltransferase	WP_043504920.1	40/54	-	-
*c2fdmP*	169	Unknown	WP_017596474.1	62/75	-	-
*c2fdmQ*	113	Monooxygenase	WP_017596454.1	65/76	-	-
*c2fdmR*	454	Biotin carboxylase	WP_017596455.1	80/87	-	-
*c2fdmS*	175	Biotin carboxyl carrier protein	WP_026120848.1	60/71	-	-
*c2fdmT*	585	Carboxyl transferase	WP_017596457.1	75/80	-	-
*c2fdmU*	111	Monooxygenase	WP_017596458.1	70/78	*fdmQ*	50/66
*c2fdmV*	113	Monooxygenase	WP_017596459.1	76/83	*fdmP*	51/70
*c2fdmW*	248	3-ketoacyl-ACP reductase	WP_017596460.1	83/92	*fdmO*	55/71
*c2fdmX*	153	Unknown	WP_015621174.1	61/74	-	-
*c2fdmY*	156	Polyketide cyclase	WP_075740187.1	67/82	*fdmI*	56/74
*c2fdmZ*	87	ACP	WP_017596463.1	46/71	-	-
*c2fdmA1*	409	KS_β_	WP_017596464.1	77/86	*fdmG*	61/72
*c2fdmB1*	422	KS_α_	WP_017596465.1	83/90	*fdmF*	63/77
*c2fdmC1*	112	Polyketide cyclase	WP_017596466.1	83/90	*fdmD*	64/76
*c2fdmD1*	254	Transcriptional regulator	WP_116247593.1	58/78	*fdmR1*	46/64
*c2fdmE1*	256	Transcriptional regulator	WP_081620746.1	67/80	-	-
*c2fdmF1*	144	Unknown	WP_017596472.1	78/89	*fdmE*	59/72
*c2fdmG1*	179	Transcriptional regulator	KPC87453.1	80/84	-	-
*c2fdmH1*	271	Serine hydrolase	WP_099880484.1	90/93	-	-
*c2fdmI1*	225	Unknown	WP_099880487.1	81/86	-	-
*c2fdmJ1*	394	Transcriptional regulator	WP_055497612.1	98/98	-	-
*c2fdmK1*	315	ACP S-malonyltransferase	WP_099880491.1	87/92	-	-

**^1^** NCBI accession numbers are given.

## Supplementary Materials

The following are available online at https://www.mdpi.com/1660-3397/18/6/284/s1; Table S1: Streptomycin MICs for *S. albus* subsp. *chlorinus* and *S. albus* subsp. *chlorinus* JR1; Table S2: Quantification of fredericamycin C_2_; Table S3: Type and location of point mutations in the genome of *S. albus* subsp. *chlorinus* JR1; Table S4: Bacterial strains and BACs used in this work; Figure S1: Calibration curve for fredericamycin C_2_ quantification; Figure S2: Important 2D NMR correlations in fredericamycin C_2_; Figure S3: HPLC-MS chromatograms of crude extracts from *S. albus* subsp. *chlorinus* JR1, *S. albus* 2P5, and its parental strain *S. albus* Del14; Figure S4: Antibacterial evaluation of fredericamycin C_2_. Figure S5: ^1^H NMR; Figure S6: ^13^C NMR; Figure S7: COSY NMR; Figure S8: HSQC NMR; Figure S9: HMBC NMR; Figure S10: ROESY NMR.
