# Supplementary material for: Novel Fredericamycin Variant Overproduced by a Streptomycin-Resistant Streptomyces albus subsp. chlorinus Strain
Source: Mar Drugs. 2020 May 28;18(6):284. doi: 10.3390/md18060284 (PMC7345809; doi:10.3390/md18060284)
Supplement: Supplementary file 1 [file marinedrugs-18-00284-s001.pdf]

marine drugs

Supplementary Information

Novel Fredericamycin Variant Overproduced by a Streptomycin-Resistant *Streptomyces albus* subsp. *chlorinus* Strain

Marta Rodríguez Estévez [1], Maksym Myronovskyi [1], Birgit Rosenkränzer, Thomas Paululat [2], Lutz Petzke [3], Jeanette Ristau [3], Andriy Luzhetskyy [1,4,*]

[1] Pharmazeutische Biotechnologie, Universität des Saarlandes, 66123 Saarbrücken, Germany; marta.rodriguezestevez@uni-saarland.de (M.R.E.); maksym.myronovskyi@uni-saarland.de (M.M.)
[2] Organische Chemie II, Universität Siegen, 57068 Siegen, Germany; paululat@chemie.uni-siegen.de
[3] BASF SE, 67056 Ludwigshafen, Germany
[4] Helmholtz-Institut für Pharmazeutische Forschung Saarland, 66123 Saarbrücken, Germany
* Correspondence: a.luzhetskyy@mx.uni-saarland.de; Tel.: +49-0681-70223

Tables

Table S1. Streptomycin MICs for *S. albus* subsp. *chlorinus* and *S. albus* subsp. *chlorinus* JR1.

Strain	MIC (µg/ml)
Streptomyces albus subsp. *chlorinus* NRRL B-24108	< 50
Streptomyces albus subsp. *chlorinus* JR1	200

Table S2. Quantification of fredericamycin C_2 produced by *S. albus* subsp. *chlorinus* JR1 and the parental strain *S. albus* subsp. *chlorinus* NRRL B-24108.

**Strain**	**Peak area (AU)**[a]	**Fredericamycin C_2 concentration (μM)**[a] [b]
Streptomyces albus subsp. *chlorinus* JR1	571,003,904	773.25 (0.1)
Streptomyces albus subsp. *chlorinus* NRRL B-24108	10,157,194	14.08 (0.0)

[a]Values indicate the average of three independent measurements

[b]Standard deviation values are shown in parentheses

Table S3. Type and location of point mutations in the genome of *S. albus* subsp. *chlorinus* JR1.

Position (bp)	Strain	Sequence (5′ → 3′)	Type of mutation
30077	WT	30068-CGGGGGGGGGA-30079	G insertion
	mutant	30068-CGGGGGGGGG**G**A-30080	
636229	WT	363224-CCCTC**C**TCGA-636233	C deletion
	mutant	363224-CCCTCTCGA-636232	
685900	WT	685893-GGCGGGGCGG-685902	G insertion
	mutant	685893-GGCGGGG**G**CGG-685903	
1120949	WT	1120943-GGCCTG**G**TAC-1120952	G deletion
	mutant	1120943-GGCCTGTAC-1120951	
1922752	WT	1922746-CCCGCG**A**GCG-1922755	A deletion
	mutant	1922746-CCCGCGGCG-1922754	
2222897	WT	2222890-GCCCCCC**C**GG-2222899	C deletion
	mutant	2222890-GCCCCCCGG-2222898	
2548236	WT	2548230-CTCCGC**G**AGC-2548239	G deletion
	mutant	2548230-CTCCGCAGC-2548238	
3714884	WT	3714886-CGACCGTGCC-3714877	C insertion
	mutant	3714886-CGA**C**CCGTGCC-3714876	
5766153	WT	5766149-GAGT**G**CGCGC-5766158	G → C substitution
	mutant	5766149-GAGT**C**CGCGC-5766158	
5766154	WT	5766149-GAGTG**C**GCGC-5766158	C → G substitution
	mutant	5766149-GAGTC**G**GCGC-5766158	
5766161	WT	5766156-CGCCG**G**CCGG-5766165	G deletion
	mutant	5766156-CGCCGCCGG-5766164	
5790341	WT	5790331-CGGGGGGGGGGT-5790342	G insertion
	mutant	5790331-CGGGGGGGGGG**G**T-5790343	
6179245	WT	6179240-GGGCC**G**CACC-6179249	G → C substitution
	mutant	6179240-GGGCC**C**CACC-6179249	
6213269	WT	6213264-TCCGC**C**GCTG-6213273	C deletion

	mutant	6213264-TCCGCGCTG-6213272	
7015221	WT	7015216-CCTTCCACCC-7015225	C deletion
	mutant	7015216-CCTTCACCC-7015224	

Table S4. Bacterial strains and BACs used in this work.

Bacterial strain	Features	Reference/Source
Streptomyces albus subsp. *chlorinus* NRRL B-24108	Strain harboring fredericamycin biosynthetic gene cluster	[2]
Streptomyces albus subsp. *chlorinus* JR1	Streptomycin-resistant mutant strain overproducing fredericamycin C_2	This work
Streptomyces albus Del14	Heterologous expression host	[3]
Streptomyces albus 2P5	*S. albus* Del14 strain harboring BAC 2P5	This work
Escherichia coli ET12567 pUB307	Donor strain for intergeneric conjugation	[4]
Escherichia coli DH10β	General cloning strain	[5]
BACs		
pSMART	AmR; BAC vector	Lucigen (USA)
2P5	BAC containing fredericamycin gene cluster	Intact Genomics (USA)

Figures

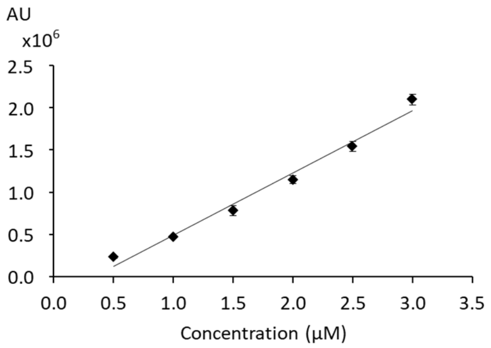

Figure S1. Calibration curve for fredericamycin C_2 quantification. Peak area is represented against fredericamycin C_2 concentration (µM). The values represent the average of three independent measurements. The black line shows the trendline that leads to the equation: $\boldsymbol{y = 7.38 * 10^5 x - 2.49 * 10^5}$.

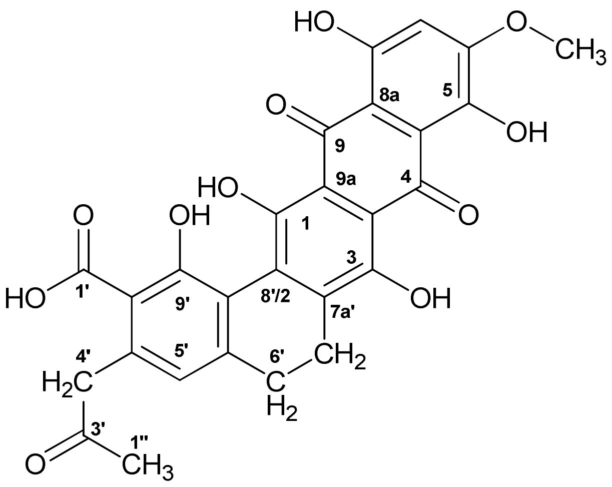

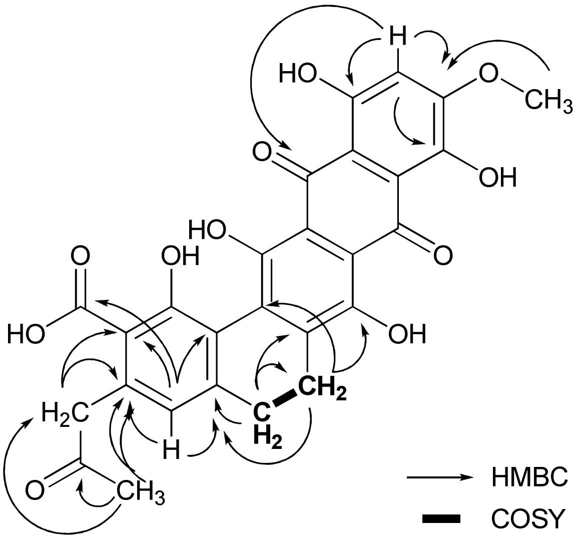

Figure S2. Important 2D NMR correlations in fredericamycin C_2.

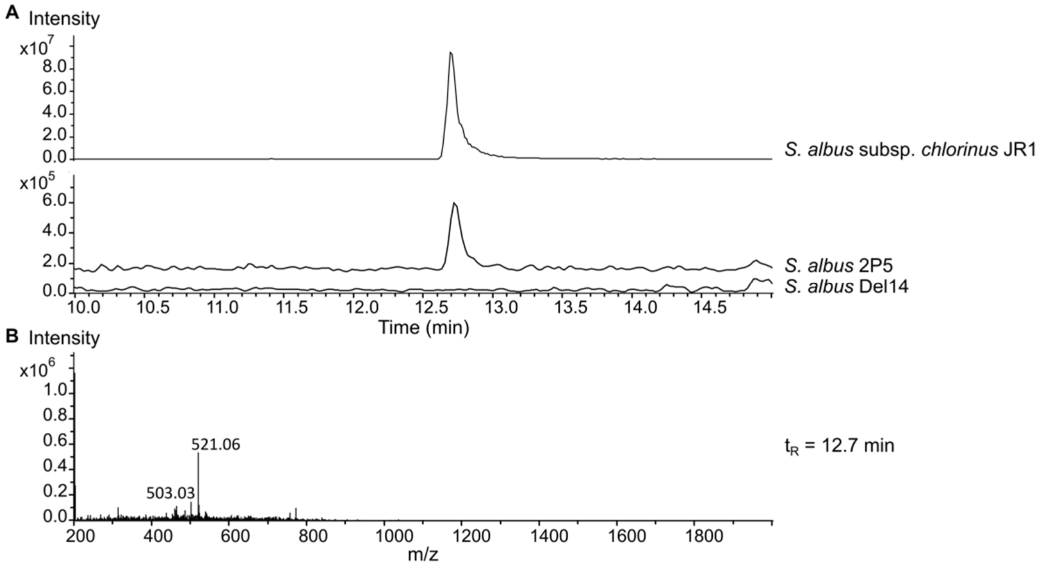

Figure S3. HPLC-MS chromatograms of crude extracts from *S. albus* subsp. *chlorinus* JR1, *S. albus* 2P5, and its parental strain *S. albus* Del14. (**A**) Extracted ion chromatograms (521.12 ± 0.1 Da). (**B**) Mass spectrum corresponding to t_R = 12.7 min from *S. albus* 2P5 chromatogram displayed in A.

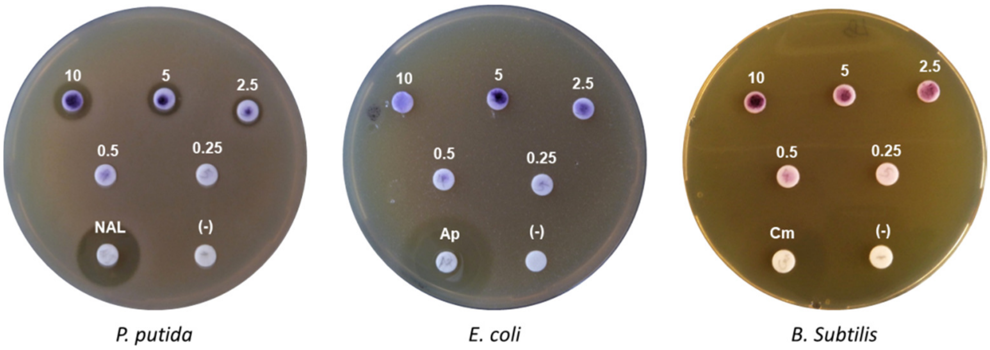

Figure S4. Antibacterial evaluation of fredericamycin C_2. Disk diffusion tests against *Pseudomonas putida*, *Escherichia coli*, and *Bacillus subtilis* are shown. Fredericamycin C_2 solved in methanol was loaded onto paper disks at concentrations of 10 mg/ml, 5, 2.5, 0.5, and 0.25 mg/ml. NAL: nalidixic acid; Ap: ampicillin; Cm: chloramphenicol; (-): negative control (methanol).

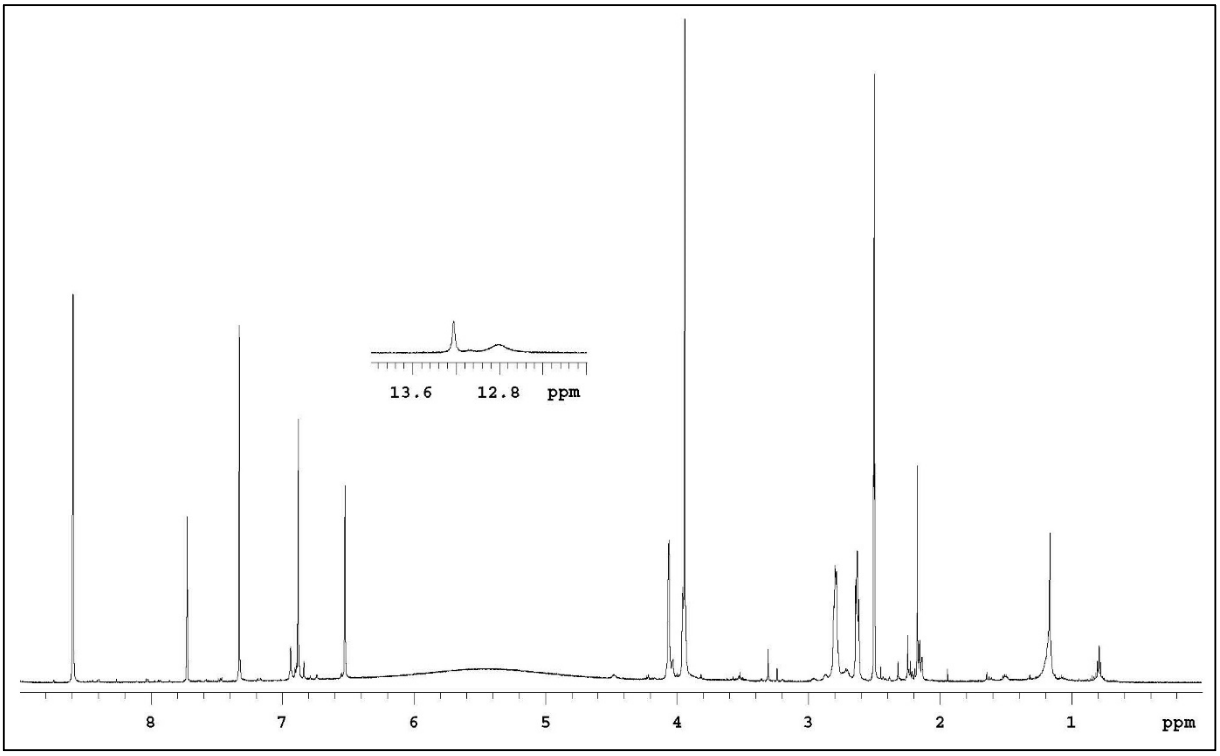

Figure S5. ^{1}H NMR (600 MHz, DMSO-d6/Pyridine-d5 95:5, 35 °C).

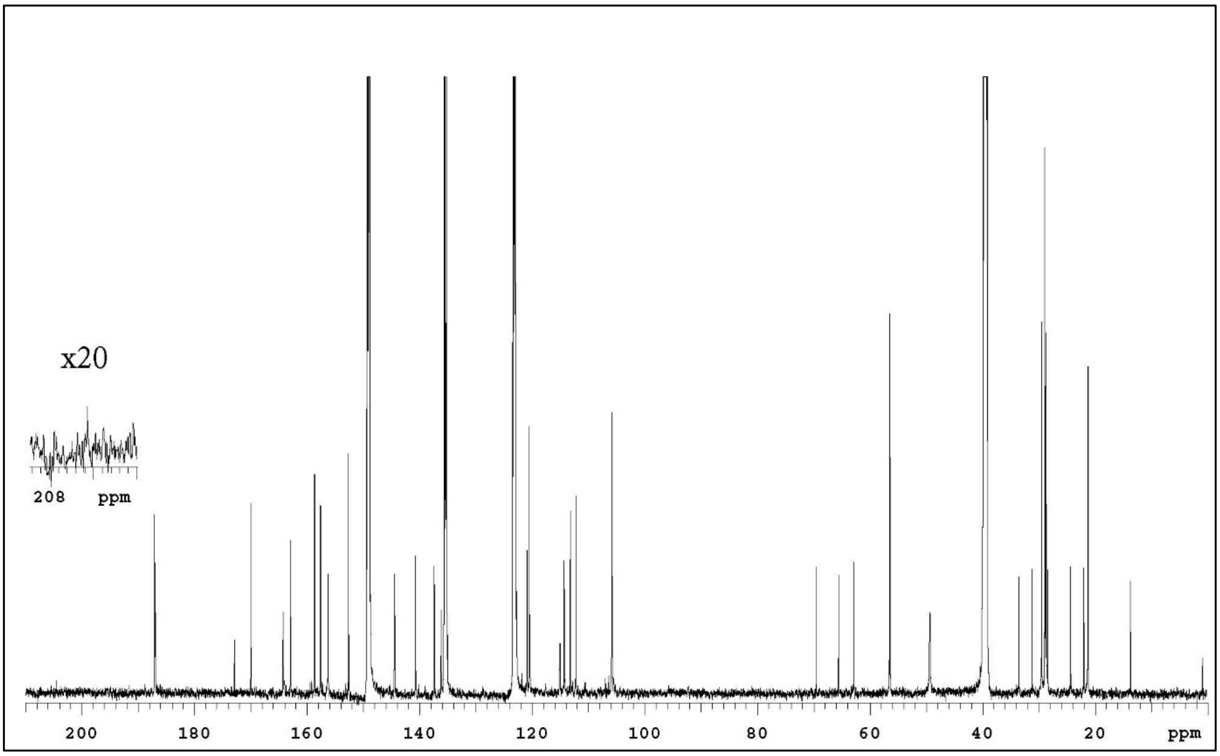

Figure S6. ^{13}C NMR (150 MHz, DMSO-d6/Pyridine-d5 95:5, 35 °C).

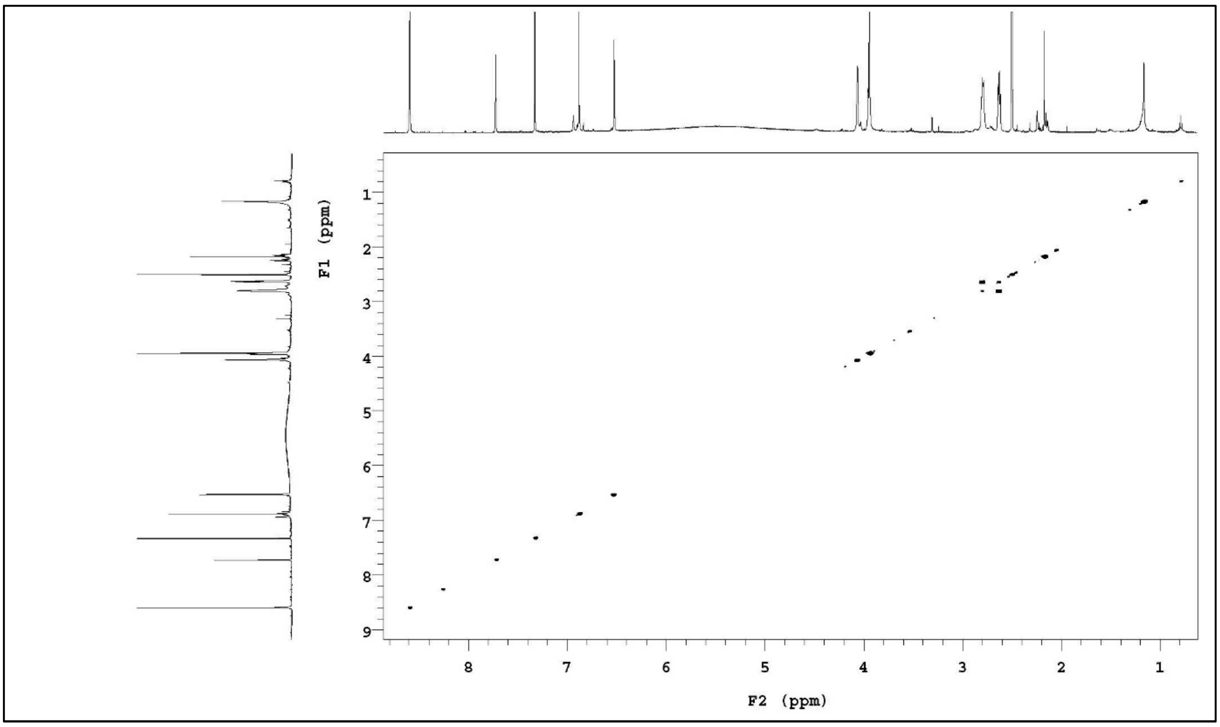

55

56 **Figure S7.** COSY NMR (600 MHz, DMSO-d6/Pyridine-d5 95:5, 35 °C).

57

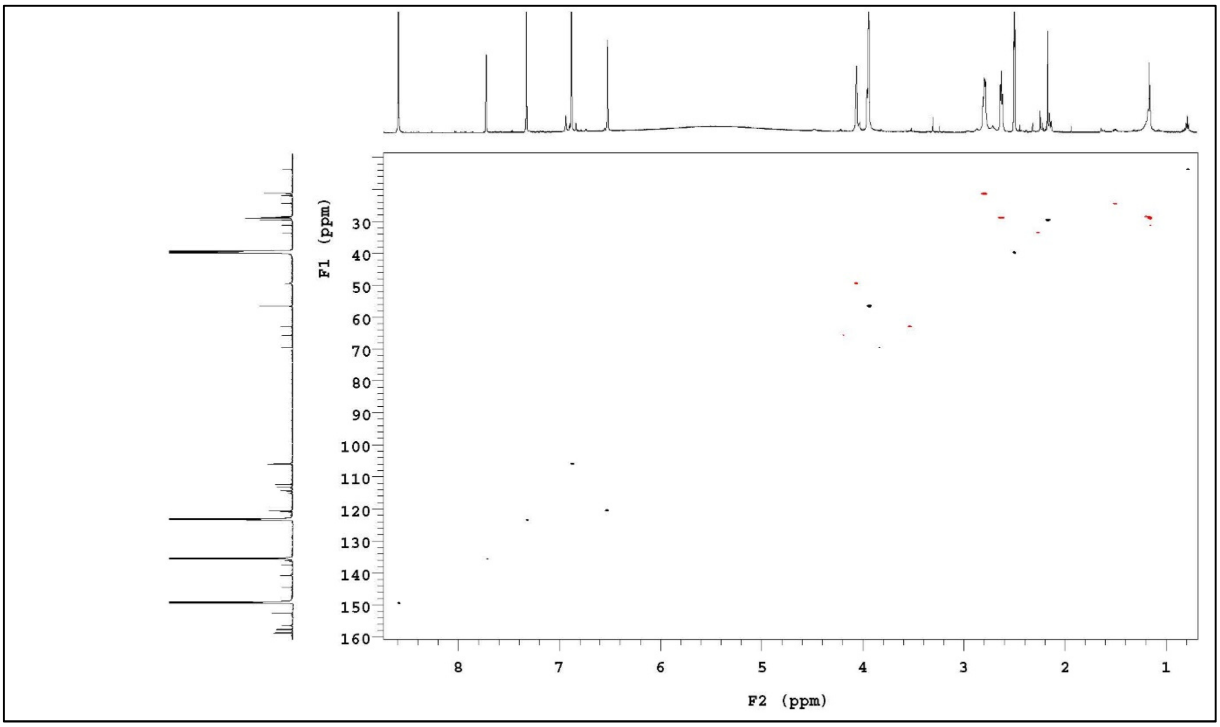

Figure S8. HSQC NMR (600 MHz, DMSO-d6/Pyridine-d5 95:5, 35 °C).

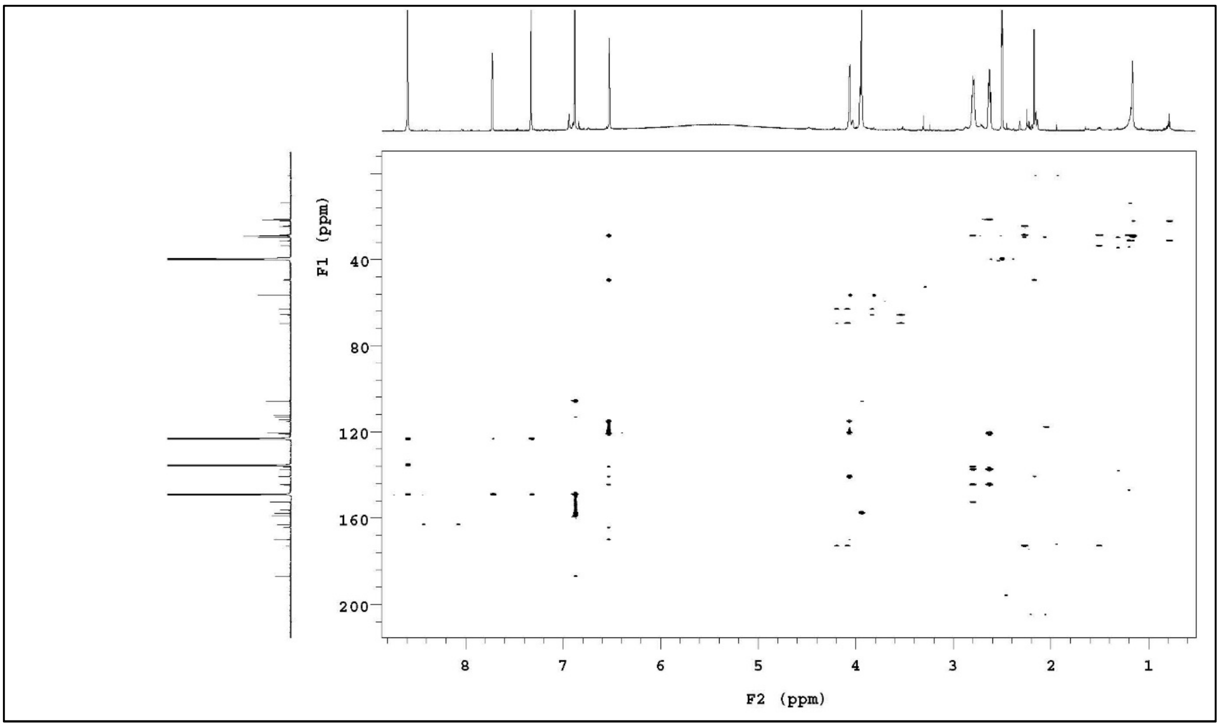

Figure S9. HMBC NMR (600 MHz, DMSO-d6/Pyridine-d5 95:5, 35 °C).

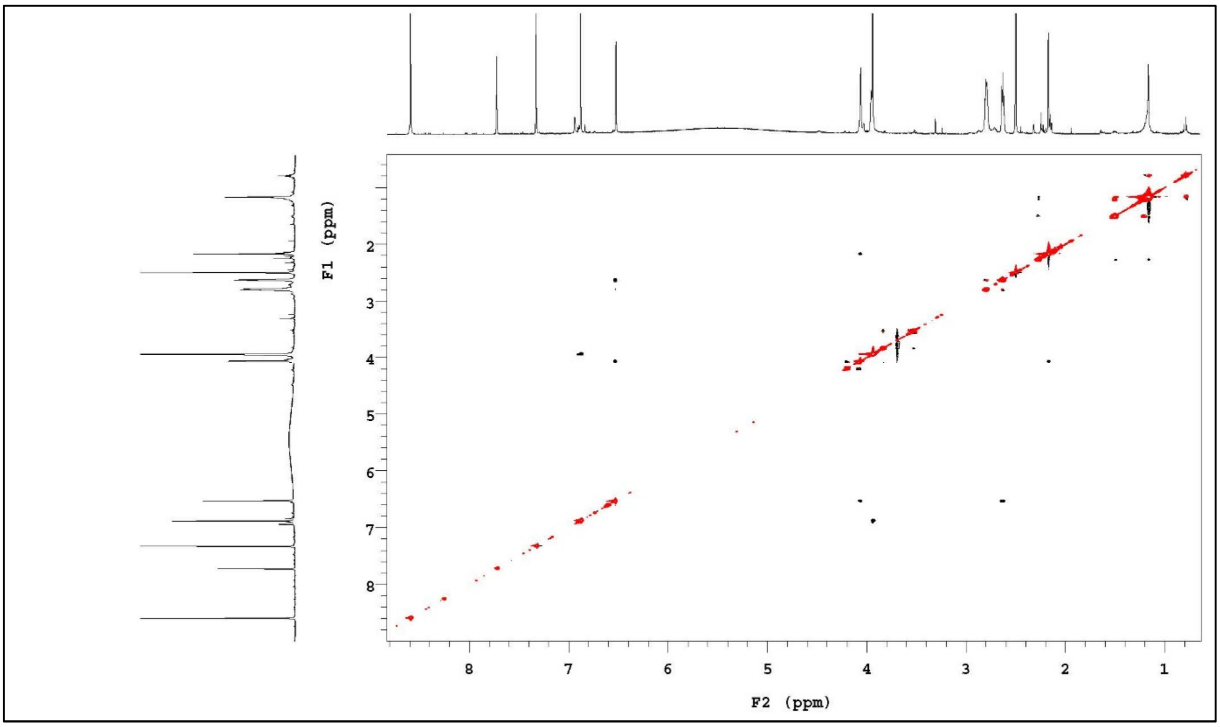

Figure S10. ROESY NMR (600 MHz, DMSO-d6/Pyridine-d5 95:5, 35 °C).

References

1. Sontag, B.; Muller, J.G.; Hansske, F.G. Fredericamycin B and C from *Streptomyces griseus*: structure elucidation after 23 years. *J. Antibiot.* **2004**, *57*, 823-828.
2. Hahn, D.R.; Graupner, P.R.; Chapin, E.; Gray, J.; Heim, D.; Gilbert, J.R.; Gerwick, B.C. Albucidin: a novel bleaching herbicide from *Streptomyces albus* subsp. *chlorinus* NRRL B-24108. *The Journal of antibiotics* **2009**, *62*, 191-194, doi:10.1038/ja.2009.11.
3. Myronovskyi, M.; Rosenkränzer, B.; Nadmid, S.; Pujic, P.; Normand, P.; Luzhetskyy, A. Generation of a cluster-free *Streptomyces albus* chassis strains for improved heterologous expression of secondary metabolite clusters. *Metab. Eng.* **2018**, 10.1016/j.ymben.2018.09.004, doi:10.1016/j.ymben.2018.09.004.
4. Flett, F.; Mersinias, V.; Smith, C.P. High efficiency intergeneric conjugal transfer of plasmid DNA from *Escherichia coli* to methyl DNA-restricting streptomycetes. *FEMS Microbiol. Lett.* **1997**, *155*, 223-229.
5. Grant, S.G.; Jessee, J.; Bloom, F.R.; Hanahan, D. Differential plasmid rescue from transgenic mouse DNAs into *Escherichia coli* methylation-restriction mutants. *Proc. Natl. Acad. Sci. USA* **1990**, *87*, 4645-4649.
